# Attentional functioning after two online mindfulness-based group interventions: Acceptance and commitment therapy and a mindfulness-based emotional regulation intervention in anxiety disorders. Preliminary results

**DOI:** 10.1192/j.eurpsy.2021.2082

**Published:** 2021-08-13

**Authors:** G. Navarro-Oliver, E. Fernández-Jiménez, I. Torrea-Araiz, T. Castellanos-Villaverde, E. Vidal-Bermejo, A. Hospital-Moreno

**Affiliations:** 1 Department Of Psychiatry, Clinical Psychology And Mental Health, La Paz University Hospital, Madrid, Spain; 2 Idipaz, Department Of Psychiatry, Clinical Psychology And Mental Health, La Paz University Hospital, Madrid, Spain

**Keywords:** acceptance and commitment therapy, Mindfulness-based Emotional Regulation, Online treatments, Attention

## Abstract

**Introduction:**

The relationship between attentional functioning and mindfulness is an intensive field of study, mainly in face-to-face interventions. However, no neuropsychological study addressed the effect of online mindfulness-based interventions on this cognitive function.

**Objectives:**

To assess changes on attentional functioning after two online mindfulness-based group interventions in adult patients with anxiety disorders.

**Methods:**

This study was carried out in a Mental Health Unit in Spain (Colmenar Viejo, Madrid). Thirteen adult patients (age mean = 51.69 years, ranging from 33 to 69 years, S.D. = 11.56) with anxiety disorders completed the interventions. The group treatments were Acceptance and Commitment Therapy and a Mindfulness-based Emotional Regulation intervention, during 8 weeks, guided by two Clinical Psychology residents. Both interventions were carried out online. The dependent variables were the scores on the TMT-A (seconds), Digit span forward and Longest digit span forward (WAIS-IV). A comparison of paired-means was conducted. Statistical significance was set at p < .05.

**Results:**

The normality assumption was met except for Longest digit span forward. The paired t-test showed statistically significant change between pre-treatment and post-treatment on TMT-A [t_(12)_= 3.81; p = 0.002; Cohen’s d = 1.056; statistical power observed = 94.0%], but not on Digit span forward (p = .45). Wilcoxon signed ranks test showed no statistically significant change on Longest digit span forward (p = .56).

**Conclusions:**

These results show a large improvement on visual attention and speed of visuomotor tracking, but not on auditive attention, after both online mindfulness-based group interventions.

**Disclosure:**

No significant relationships.

